# Trust in health care encounters and systems: a case study of British pensioners living in Spain

**DOI:** 10.1111/1467-9566.12163

**Published:** 2014-12-03

**Authors:** Helena Legido-Quigley, Martin McKee, Judith Green

**Affiliations:** Faculty of Public Health and Policy, London School of Hygiene and Tropical Medicine

**Keywords:** trust, migration, older people, habitus, Spain

## Abstract

Research on trust in health care faces two enduring challenges. Firstly, there are conceptual ambiguities in distinguishing trust from related concepts, such as confidence or dependence. Second, the tacit understandings which underpin the ‘faith’ element of trust are difficult to explicate. A case study of British pensioners who have moved to Spain provides an opportunity to explore trust in a setting where they often have a choice of where to access health care (UK or Spain), and are therefore not in a state of dependence, and in which the ‘differences’ of a new field generates reflection on their tacit expectations of providers and systems. In accounting for decisions to use (or not to use) Spanish health care, British pensioners cited experiential knowledge of symbolic indicators of trustworthy institutions (they were hygienic, modern, efficient), which contributed to background confidence in the system, and interpersonal qualities of practitioners (respect for older people, embodied empathy and reciprocity) which evoked familiar relations, within which faith is implicit. In contrast, with limited recent access to the British system, their background confidence had been compromised by reports of poor performance, with few opportunities to rebuild the interrelational bases of trust.

## Introduction: trust in health care

Luhmann (1988) noted the neglect of trust in classical sociological theory and suggested that empirical work has consequently tended to utilise common-sense notions of confidence, or positive attitudes, which risk merely reconstructing routine platitudes about the need to recognise social relationships, or solidarity, in modern societies. However, over the last two decades, a growing body of theoretical and empirical research has attempted to move beyond merely studying common-sense evocations of trust in descriptions. This has helped both to conceptualise trust and to address questions of what functions it plays in particular kinds of social organisation (see Mistzal 1996 for an overview). Möllering, for instance, draws on Georg Simmel's (1950) attempt to delineate the necessary conditions for a relationship in which one actor has confidence in another, whether that other is a person or an institution (Möllering 2001). This confidence, argued Simmel, arises both from prior knowledge about the other, and also from a rather less easy to identify quality: a more mysterious sense of faith in the other. The necessity for faith, what Möllering (2001) calls suspension, means that trust cannot be reduced to a rational calculation by one actor of the ethical motivations or competence of another. Pragmatically, difficulties in conceptualising the more esoteric elements of trust mean that they are difficult to research, being rooted in tacit knowledge and taken-for-granted assumptions that can be difficult both to recognise and explicate. Gambetta's (1988) generic definition, for instance, incorporates the rational assessment of expectations, but not the more ambiguous sense of faith:

Trust (or, symmetrically, distrust) is a particular level of the subjective probability with which an agent assesses that another agent or group of agents will perform a particular action, both *before* he can monitor such action (or independently of his capacity ever to be able to monitor it) *and* in a context in which it affects *his own* action. (Gambetta, 1988: 217).

For Luhmann (1988), the key to understanding trust as a specific concept in modern society was to recognise that it results from particular articulations of environments and systems. It can be distinguished from concepts such as familiarity and confidence in that it results from relationships in which it is (reasonably) possible for the actor to assess the future risks and benefits of an action and to choose whether to engage in it. A relationship of trust is therefore one that presupposes an understanding of risk (as opposed to danger) and one in which individuals are both invested in a future (in order to take risks), and in a position to make a choice. Confidence, on the other hand, results from expectations that may be disappointed, but which the actor cannot, reasonably, avoid and which thus do not generate any particular anxiety: in that, for instance, medical professionals are appropriately qualified, or that the health insurance company will exist tomorrow. Trust becomes salient in situations when there is a realistic choice to make: perhaps to undergo an operation where the risks and benefits are uncertain, or to choose one health-care system over another. Meyer and Ward (2013), in considering the case of patients at high risk, suggest extending Luhmann's distinctions to include dependence, to describe situations in which patients have no option but to trust.

Both empirical research and theory on trust in health care make a distinction between interpersonal trust, relating to encounters of individual patients and health-care professionals, and institutional trust, relating to health-care organisations or systems (Calnan and Rowe 2008a, Goold and Klipp 2002, Luhmann 1988, Mechanic 1998). The vulnerability of the patient within a Parsonian model of the doctor–patient relationship is core to many conceptualisations of interpersonal trust, as in Hall *et al*.'s definition: ‘the *optimistic* acceptance of a *vulnerable* situation in which the truster believes that the trustee will *care* for the truster's interest’ (Hall, *et al*. 2001: 615, emphasis in original). With a recognition of the dual nature of trust (a rational component based on judgments and beliefs and an emotional component based on affective relationships), Calnan and Rowe suggest three factors that make trust important at the level of the encounter: the vulnerability associated with being ill; the asymmetry of information between the two parties and the uncertainty about the intentions and competence of health-care professionals (Calnan and Rowe 2006, 2008a).

The second level, that of trust in the system, has perhaps been less intensively researched (Calnan and Rowe 2008b, Goold and Klipp 2002). Here, what is termed trust in research is often more akin to what is, in Luhmann's conceptualisation, confidence. Giddens (1990), for instance, holds trust in abstract systems to be a generic feature of late modern society, in which citizens are unlikely to have much understanding of expert systems such as financial markets, complex technologies or governance structures, and must therefore simply trust that these work. Arguably, as citizens are required to become more expert and reflexive about their health-care choices, the requirements for routine confidence in health systems decline and those for trust expand.

How trust in the system relates to trust in the individual professional is not straightforward, and a range of articulations have been suggested in the literature. Gilson (2003), for instance, argues that trust has come to the fore as a research topic in part as a result of recognition of the inherently interpersonal nature of health-care systems: trust in the system, in this view, is to some extent a function of trust in providers within that system. In a later discussion of building trust in health-care systems in low- and middle- income countries, Gilson and colleagues suggest, therefore, that interventions at the level of health worker behaviour are likely to enhance trust in the system (Gilson *et al*. 2005). Alternatively, Mechanic and Meyer (2000) suggest a different causal direction, in suggesting that declining public faith in Health Maintenance Organisations in the USA potentially spills over to damage trust in individual providers, if patients lose faith in their physicians’ ability to put their interests first.

However, as Hall *et al*. (2001) have noted, trust in a known care provider may well have different foundations from trust in systems and, following Luhmann, we might expect that trust in individual practitioners would articulate, but not necessarily correlate, with trust in systems. This is certainly borne out by empirical work in other areas, which suggests that criteria for assessing trust may operate differently at individual and system levels. On food systems in Europe, for instance, there is evidence that confidence in institutions that protect food quality is greatest in those most distant from the actor, and consequently local actors are often not trusted. In the interpersonal sphere, however, it is the most local food providers that are more trusted (Green *et al*. 2003). There is, then, a remaining empirical question about how interpersonal trust and institutional trust in health-care systems interrelate.

One potential way of incorporating the interpersonal and system levels is to draw on Bourdieu's (1977) concepts of habitus, field and doxa. In Bourdieu's words, habitus can be considered: ‘systems of durable, transposable dispositions, structured structures predisposed to function as structuring structures’ (Bourdieu 1977: 72). Although deeply rooted, these dispositions are flexible: not rules, but strategies, with the possibilities of (within limits) improvisation. If different health-care systems can be considered social fields of practice, each with its own set of structures and scripts, which form the conditions of possibility for particular understandings and actions, then doxa, the taken-for-granted understanding of what is going on, will result from the interrelationships of habitus and field. Where habitus is most closely aligned with field – for instance, in contexts where patients are utilising an enduring health-care system with which they are familiar – then the sphere of doxa, that which is taken-for-granted, is likely to be difficult to access in research, simply because it relates to practical comprehension, which is by its nature unremarkable. As Luhmann (1988) suggests, when asked to account for trust, actors are likely to provide reasons (a theory of practice, in Bourdieu's terms), but these reasons may not reflect the more tacit assumptions about why trust is, or is not, bestowed (a logic of practice). In contexts where field and habitus are less well aligned – when encountering a new health-care system, for instance, – a space for observing these logics of practice potentially opens up, as actors’ tacit assumptions are challenged.

## The case study: British pensioners living in Spain

A study of British pensioners who have moved to Spain provided an opportunity to explore the conditions for trust in a case where health-care users were encountering a new field. We have previously shown (Legido-Quigley *et al.,* 2012, Legido-Quigley and McKee 2012) how, when pensioners move to Spain, they have to navigate new social fields in the destination country, consisting of social networks linking back to Britain, a specific expatriate network in Spain and what they called a Spanish way of life, which includes changes in diet, activities and modes of engagement with the public sector. This article focuses on the health-care system as one such new field, in which acquired dispositions, from previous experience with the National Health Service (NHS) in the UK, might come into conflict. It is not known exactly how many pensioners from the UK have moved to Spain: estimates of the number of British residents aged over 65 in Spain of around 125,000 (Instituto Nacional de Estadistica 2014) are based on those formally registered with the municipality and do not include large numbers of people who spend part of their time in each country. Once registered, British pensioners are entitled to access the Spanish National Health System (SNHS), which (like the British NHS) at the time of this study provided a universal health-care system, paid for largely by general taxation and free at the point of delivery (Durán *et al*. 2006). Both the NHS and the SNHS are accessed through a comprehensive primary care system which performs a gate-keeping role, and in which practitioners are paid a combination of salary and capitation. In Spain, a small private primary care sector provides fee-for-service care (Durán *et al*. 2006).

## Methods

This article draws on an analysis of data generated in a qualitative study conducted between April 2008 and August 2009, which aimed to explore the health-care experiences of older British adults living in one of two Spanish autonomous communities for at least 3 months a year (Legido-Quigley *et al*. 2012). The autonomous communities were chosen for their contrasting migration histories. In the first, Valencia, most retirees moved to Spain in the early 1990s, typically to live in large urban areas around the Costa Blanca and Costa del Sol where most expatriates are from the UK (Casado-Díaz 2006, Rodríguez *et al*.^1998^). In the second, the Autonomous Community of Baleares, migration began much earlier, in the 1970s, with a majority of German expatriates and fewer living in large urban concentrations (Salvá 2005). In these two regions, participants were sampled iteratively and purposively for maximum variation in terms of retirement experiences, time since migration, residence type, links with expatriate community, health status and health-care experiences. They ranged in age from those who had taken early retirement in their fifties to those who were now over 90, and from recent migrants to those with 25 years’ residence. Participants were identified using a range of strategies, including respondents to a recent household survey that had sought to identify international retirees (in Valencia), expatriate organisations and social events, the identification of British names in telephone directories and, in remote villages, the use of Spanish intermediaries, such as shopkeepers, to identify English residents. A total of 62 people participated in 41 interviews, 20 with a single person and 21 with couples (see Table1), including four interviews with individuals who had initially retired to Spain but had since returned to the UK.

**Table 1:** Place of residence by gender, age and years lived in Spain

Residence	Gender		Age range				Years lived in Spain				
	F	M	50–59	60–69	70–79	>80	0–5	6- 10	11–15	>16	Total
Valencia	17	18	3	17	14	1	7	17	9	2	35
Mallorca	16	7	3	8	9	3	3	10	2	8	23
UK	2	2	0	2	2	0	2	2	0	0	4
Total	35	27	6	27	25	4	12	29	11	10	62

All interviews took a narrative form, with a topic guide covering reasons for migrating, family relations, everyday life and lifestyle, health-care needs, health-care experiences, experience of other services and future plans. Most interviews lasted over an hour, as participants were willing to talk at length about their lives and health experiences. Most interviews were undertaken by the first author, who has dual Spanish/English nationality, with the third author attending some and five undertaken by an older, English interviewer. In this article, all names are pseudonyms and identifying data have been removed to maintain confidentiality. Analysis of the data used elements of the grounded theory approach (Strauss 1987), including initial line-by-line analysis of early interviews to develop a coding frame and the use of comparisons in the dataset and deviant cases to test and develop emerging hypotheses.

In this article, we focus on the accounts that British expatriate pensioners provided in interviews of using, or intending to use, British and Spanish health-care services. Trust was not a specific topic of the interview, but emerged as a theme in the analysis. Rather than relying solely on what they said about trust in health care, we have exploited the advantages of this case to infer, from accounts, the conditions for trust. In accounting for their decisions, British pensioners make both explicit and implicit comparisons with the health-care system they previously used (in the UK), and in some cases were still using. Most pensioners were in a position of choice, being able to return to the UK to receive health care, or (for some) to utilise the private sector. The decision to use, or intention to use, one system could therefore be considered, in Luhmann's terms, a situation that demonstrated trust, as there was typically a choice to be made.

## Findings

### The puzzle of trust in the Spanish health-care system

In general, British pensioners reported high satisfaction with Spanish health-care services. In discussing their satisfaction, trust emerged as a key theme, both as an *in vivo* shorthand code to describe orientations to health-care provision, but also as a puzzle in the data, in that trust was on occasion bestowed when either there was little direct experience to draw on, or even when things had gone wrong. Ronald and Judy, for instance, had been in Spain for 14 years, having retired early. Like many who retired to Spain, they had moved for the climate and lifestyle, and reported that health-care provision in their destination country had not been a consideration: it was not, they said, ‘a tick that we had on any of our boxes – we were young and healthy, we just didn't consider it’. To date, their demands on the Spanish health-care system had been minimal, only involving some private dental care. However, when asked where, if they needed an operation, they would like it to be done, in the UK or Spain, Ronald replies without hesitation: ‘Well, obviously I would prefer to be in Spain’.

This ‘obviously’ may reflect no more than commitment (or a rationalisation of commitment) to a new life in a new country, where health care, like the rest of life, was likely to be ‘better’: perhaps a clear statement of faith, rather than a calculation of risks and benefits, for a couple with little direct experience on which to make rational assessments. However, similar responses were not only common across the data set, but were also voiced in sharp contrast to the brief, but usually dismissive, assessments provided of other Spanish institutions, such as local town halls, where a residence card had to be applied for, or the police service. Unlike health-care institutions, these were almost universally described as complex, bureaucratic and inefficient. Thus, trust in the Spanish health-care system (which one would ‘obviously’ use if needed) did not appear to be simply a routine statement of migrants’ confidence in the public sphere of a new home, compared with the one left behind. Mark, for instance, who had considerable experience of the health-care system both for himself and his wife, who had died some 7 years previously, is typical in that his positive assessment of health services is not generalisable to other aspects of the Spanish public sector:

I go to the new hospital … it's the best hospital in Europe … it's beautiful, its clean, its airy, it plays music in the corridors. … Can't fault the medical out here, the attention you get is marvellous, it's *one thing* they really have cracked (Mark).

Mark's assessment may simply reflect a rational calculation of superior health-care facilities, but similar expressed preferences for the Spanish health-care system were typical, even for interviewees who reported experiencing challenges in accessing the system and for those whose personal experiences had been less than satisfactory. Challenges reported included having to pay for translators, as few interviewees had learnt enough Spanish to manage a health-care encounter, finding transport to the hospital (especially in more rural areas) and managing in-patient personal care and after-care, given that in the Spanish system, family members are expected to carry out many of the tasks that British pensioners would have expected (from their experiences of the British NHS) nursing staff to undertake. However, these challenges, and even the occasional more severe shortcomings, rarely undermined faith in either the system or individual professionals.

Andrew and Rosemary, for instance, a couple in their seventies who had moved to Spain 11 years previously, tell a complicated story of Andrew's various eye operations in the SNHS, a story that spanned some 4 years, beginning with delays in referral, and including one operation reportedly carried out on the wrong eye: ‘they operated on the blind eye, which was useless!’ They considered moving to the private system but were reassured by the surgeon, who then carried out what the couple describe as: ‘the most complicated eye operation that [they had done] in the hospital’. The various hospital visits had been troublesome administratively, as they struggled with the registration system, and costly, with the need to pay for Spanish translators, sometimes for whole days as they travelled to and waited for appointments. However, perhaps surprisingly, given these problems, the couple's story was punctuated by frequent positive asides about the system in general in Spain. As the story ends, the interviewer asks ‘Were you ever tempted to go back to London for the operation?’ Their reply is telling:

Rosemary: Not bloody likely! [laughs] We would probably come back with having his head off! … I would sooner go to a local butcher…
Andrew: I reckon operations here are very good.

Similarly, Deborah, who had moved to Spain 14 years previously, recounted a poor experience visiting a friend recovering from an operation, who had been left ‘to their family to look after them during the night’ and a misdiagnosis of her own back problem by a local doctor. She, too, concludes with positive comments about both the system in general, and the individual provider who had misdiagnosed her back problem, noting ‘that wouldn't stop me going back to the doctor again’.

Here, then, is apparent evidence of faith, given that intentions to use Spanish health care apparently arise despite, rather than because of, rational calculations of risks, benefits and costs. Further, the robust criticisms of other aspects of the Spanish state suggest this is not merely courtesy bias from participants answering questions from a Spanish-identified interviewer. Neither does it straightforwardly represent mere dependence (Meyer and Ward 2013), a need to trust, or to rationalise current choices to minimise regret because there are no other options: participants such as Rosemary and Andrew were not unusual in having both the financial and other means to return home for treatment if necessary, or to pay for private care. That Spain, for many participants, would be the ‘obvious’ choice if future health needs arose, even if other options were available or (on occasion) when atrocity stories could be told about things that had gone wrong, suggests a degree of what could properly be called trust. To unpack the foundations of this trust, we turn first to the level of the encounter.

## Respect and reciprocity: the ageing habitus in a new field

At the level of the individual encounter, it has been argued that a decline in automatic deferential trust in experts means that trust increasingly has to be earned afresh each time and is predicated on the quality of interpersonal relationships (Brown 2008, Giddens 1990). In their encounters with health providers, many participants in this study reported not only that they experienced caring attitudes by professionals in the Spanish system, but that these demonstrations of caring were both exceptional (‘beyond the call of duty’) and unexpected. Drawing on experiences with his wife's cancer specialist, Mark explicitly answers a question about confidence in the system with a reference to the interpersonal skills of their consultant:

Interviewer: Is there anything else … about the health-care system?
Mark: I've yet to fault it [… the health-care system has] been very good for me and it's been very good for my wife. She had confidence in it and that … It says a lot because if you are ill you've got confidence, the specialist she had … he was very good, a good bedside manner, I can't fault him.

Although a good bedside manner might be a routine expectation of professionals, Mark describes the level of caring as beyond expectations. One unexpected element that he (and many other participants) noted was *embodied* empathy (Brown *et al*. 2011) material demonstrations that health-care professionals cared about the patient as a person:

The nurses, how many nurses turn around and give a patient a hug? And yet here they do…. They made a terrific fuss of her, you don't get that in England, you don't get a hug or anything. (Mark)

Other elements mentioned as unexpected in the dataset included the use of first names, doctors sharing stories of their own experiences in the consultation and treating the patient like family or with respect. That these features were notable suggests that habitus was somewhat misaligned with the new field. This was particularly evident around an ageing habitus that, for British pensioners, was one aligned to expectations of social marginalisation and dislocation from family. In Spain, reportedly, new rules of the game were encountered, with not only Spanish families but also in wider society, conferring status on older people: ‘You come here, [and you see] the standard the elders get from the families’ (Douglas). Interactive elements of health-care encounters disrupted expectations of marginalisation, with health-care providers, cleaners and receptionists, reportedly treating participants in ways that were out of the ordinary, given their normative expectations of an ageing habitus. Rebecca, for instance, was in her seventies and had been living in Spain for 26 years. Her husband had died in hospital after a stroke and she recalls:

They made him as comfortable as possible and they were very kind to him … I couldn't fault it … even the man at the desk said ‘I am very sorry Señora’ … In my estimation you wouldn't get better treatment. (Rebecca)

As Rebecca goes on to say: ‘I mean, sometimes, when people are older they are not so bothered with them, but they couldn't have been kinder’.

Assumptions about patients' roles were also disrupted by a similarly unexpected reciprocity in health-care encounters, with participants reporting being addressed by their first names, and consulting with doctors who shared stories of their own experiences. One interactive accomplishment of this reciprocity was that the patient–professional relationship was at times rhetorically constructed as a family-like relationship, rather than simply a service-orientated one. In her account of her husband's interaction with his doctor, Ruth, for example, utilises these familial references as a rationale for both his and her own faith:

And Robert said to him ‘Yes, but I've got confidence in you’ and he was a lovely man and he treated, well his own father had had cancer and he treated Robert almost like a surrogate father. (Ruth)

Familial relations are ones within which obligations go beyond the service encounter, and ones in which the foundations of trust relate to familiarity. In Luhmann's (1988) terms, this is an example of the symbolic force of familiar terms (such as father) in an unfamiliar sphere. Until disappointed, such symbolic relationships foster faith because they reframe the system as one in which risk assessments would be inappropriate: trust is implicit.

## Communication: making an effort and its limits

Establishing the communicative elements necessary for faith in the encounter was a challenge, given that British pensioners (in general) had very limited skills in Spanish languages. It is therefore not surprising that communication was identified in several cases as a key contributor to claims of trust, and its absence as a reported reason for trust breaking down. A common *in vivo* code was that of ‘making an effort’ to describe health-care providers’ attempts to communicate despite a language barrier. Making an effort was described in actions such as attempting to speak English, taking time to conduct the consultation with the aid of dictionaries, patience, and the use of non-verbal communication such as physical contact (as above), smiling and mime. ‘To be honest most of the doctors speak English. I've got a lady doctor, and she is lovely. She speaks, she tries to speak English more than Spanish’ (Mark).

The fact that health-care professionals were trying hard to communicate, with displays of kindness, respect and reciprocity, signalled (for participants) that the provider favoured building a human relationship in which the patient could trust that they had their best interests at heart. The importance of such visible effort-making was evident when it was absent, and trusting relationships could not be established. In the few deviant cases where there was a reported reluctance to use Spanish health care, communication difficulties were often cited as an issue. In these cases, the lack of making an effort was not simply a pragmatic barrier to access but also a barrier to the establishment of trust. Kenneth and Joyce, not yet of state retirement age, for instance, recalled several stories of friends who had had less than satisfactory encounters with the Spanish public system, including complaints about the lack of privacy in hospital wards and lack of aftercare. They were unusual in that they also said: ‘If there was a major problem, we would go back to the UK’. It transpired that finding health providers who had made an effort had been a problem for them:

We haven't registered with the local doctor here in [town name], because here they speak only Spanish, they won't entertain speaking English … the receptionist can be a little bit awkward … she insists you speak in Spanish. (Kenneth)

The effects of the absence of effort-making were also evident in some accounts of professional interpreters: they may be rationally trusted to do a competent job but, given that the task was undertaken in an exclusively service relationship, the faith element indicated by making an effort could not be demonstrated. As one recently arrived resident said of professional interpreters: ‘Well, certainly, the English ones … they jump on the band-wagon and you know it's an opportunity for them to rip off their own people, isn't it?’ (Andrew)

A second important deviant case was long-term care, such as rehabilitative or palliative care. Given the new rules of the game, which emphasised the importance of family, this was widely noted as being less well provided in Spain than in the UK. Vera, for instance, who had been living in Spain since 1970 and whose Spanish was good enough to manage health-care encounters, was unusual in that she lived in one of the few available residential care homes for older adults. Her choice not to go back to the UK for care had been, she said, largely a financial one, although she also preferred the central village location of her residence in Mallorca, compared with the relatively isolated UK facilities she had seen. However, when recalling her mother's death from cancer some years earlier, Vera notes that communication barriers were a contributor to the decision to take her mother back to the UK:

She didn't learn the language and so there was, if she was going to have to have to be nursed, and final nursing, it would have been hard for her in a foreign language. So we took her back to Britain. We thought, you know, she was obviously going to need final care. (Vera)

Making an effort may be sufficient to mark the health-care provider's inherent trustworthiness, and that they are orientated towards the patient as an individual, but it is not, in itself, it seems, a sufficient criteria for trust in situations of high dependence.

## The person in the system

If communicative actions in the encounter signalled the provider's orientation to the patient's individual needs and evoked family-like settings in which trust is the default position, other reported features of health-care systems were used more explicitly as rationales for bestowing trust, as they suggested calculable indicators of needs being prioritised. Time, for instance, was a commodity that could be allocated to individuals in excess of the system's rules, as Jacqueline explains:

They make the appointments 7 minutes apart. Well, he [the doctor] spends 20, 25 minutes with every person and it happens every time. … But, you think, well, you know, he's doing it to everyone so you're gaining extra care. (Jacqueline)

Similarly, receiving thorough care was on occasion offered as evidence that Spanish health professionals were not worried about the implications of requesting multiple tests and recommending expensive procedures. That time or thoroughness could be rationales for trust was seen in the contrasts made between experiences of the Spanish system and experiences of (or, more often, assumptions about) the health-care system in the UK. Dorothy, for instance, who had been living ‘on and off’ in Spain for many years, but full time for only 2 years since early retirement, cites her comparative expectations of the British NHS as a rationale for trusting the thoroughness of care in Spain:

In England they would probably give you a blood test and couldn't find anything, so they won't do anything … I think in the UK there is a reluctance to, the doctors, I think, are more conscious of the cost of the drugs which they are looking to prescribe; therefore they won't always give you, you know, the drug which might be the best one for you. (Dorothy)

The key factors enabling faith at the level of a health-care encounter were, then, those of embodied demonstrations of caring, familial-type relations and indicators that the patients' individual interests (for time, thoroughness) would be prioritised ahead of those of the system. To some extent these factors were articulated, as in Dorothy's account, as rational assessments through contrasts with practitioners in other systems (in the UK) which could no longer prioritise the individual. However, it is not clear how these factors can underpin trust in the system itself. Indeed, a system in which personal patronage and familial-style obligations outweigh the institutional demands of fairness or efficiency would be one in which, in theory, routine confidence might be diminished. The centrality of family relations is one example. Although a positive element of the health-care encounter, the gap between habitus and field generated practical challenges for patients at the system level, with the expectation that family would provide personal care in hospital and on recovery. Widely commented on as a negative feature (‘the one downside’) of Spanish health care, this was misaligned with pensioners’ own habitus, particularly given their social field of dispersed family relations. However, as a sequel of a new, and on the whole, more positive, set of dispositions about the role of the family, this apparently remained an irritant, rather than undermining trust in the health-care system as whole.

## Institutional indicators of a trustworthy system

What, then, were the conditions that made possible trust in the system, such that most participants would hypothetically choose the Spanish system over a return to the UK? At the level of the abstract system, it is not clear that participants in this study could be described as trusting. Indeed, many were extremely vague about the system, commenting on not only their lack of understanding about age or contributory entitlements to various kinds of health care, but also on occasion on their lack of access to information. However, in practice, few had failed to access the health services they needed or, as we have described, to trust them as a preferred option for health care. This is perhaps a case of confidence that the system functioned, even if it was not clear how. What was trusted, more specifically, were health-care institutions, whether they were the local primary care providers or the many large and small hospitals referred to in participants’ stories. These institutional points of encounter with the system provided participants with experiential evidence of not only a working system, but one which epitomised what a health-care system *should* look like. The levels of cleanliness encountered were perhaps the most striking example of this. Rosemary, for instance, describes a local hospital thus:

You could eat off the toilet floor. It's spotless, absolutely A1. If I had to give 100, out of 100, I would give them 110%. You cannot fault the hospitals.

She goes on to say: ‘The place is like a clinic’. To describe a hospital as like a clinic suggests the symbolic nature of a hygienic aesthetic: clinical refers to both the medical field, but also to a style – one of clean, uncluttered and unemotional efficiency. The elision of aesthetic and medical connotations of hygiene is also evident in Kathryn's citation of high levels of cleanliness as not just a corollary of, but as an explanation for, her assessment of treatment quality:

The treatment is excellent … you know the floors are clean, I know they are all marble floors, but they are all so clean. I was waiting for a X-ray for something one day and a woman came round sweeping three times while I was waiting and in the space of two hours she did a round three times. Which in England is once a day. (Kathryn)

Symmetrically, a lack of cleanliness was a frequently mentioned (although not the only) reason for distrusting the UK system. Like Kathryn's, most of these comparisons were in unelaborated comments made in passing, although Rosemary's comparison of the outstanding cleanliness of Spanish hospitals was in sharp contrast to her memory of a UK hospital in which she visited her sister:

I had to actually call the cleaning supervisor in to clean my sister's room. It was filthy and it still wasn't done the next day and I had to go in with wash cloths and do the window sills, underneath the bed, in the sinks, everywhere cleaning and polishing. I had to do the lot. The hospitals in England are terrible, disgusting.

Like cleanliness, modernity was evoked as shorthand rationale for trusting institutions, with several comments about the availability of, for instance, the ‘latest equipment’ or automated reception systems. When discussing these rationales, the sources drawn on were almost always experiential evidence, for those who had resided in Spain for more than a few years, or reported accounts from known others, such as expatriate friends. Indeed, the importance of experiential evidence for making these assessments was underscored by the occasional exhortation for the interviewers to see for themselves: Rosemary, quoted above, completed her eulogy to the cleanliness of the local facilities by telling the interviewer to ‘see it while you can … you'd be gobsmacked’. Although on occasion contrasting comments on the British NHS also referenced personal experiences (as in Rosemary's account, above), more frequently they utilised two other source of evidence that were not apparent in accounts of the Spanish system. One was British media reports:

There are people in the UK who have had problems being on the National Health and it is such a waiting list, they have died while they were waiting. It's been on television many times, hasn't it? (Kenneth)

The second source was a general reference to the common stock of ‘what everybody knows’. Dorothy, for instance, in contrasting Spanish and English approaches to ordering tests, tellingly couches her account of England in speculative terms (‘they would probably … I think’). Similarly, Margaret, in recalling two hip operations in Spain, which followed problems in getting a good diagnosis and appropriate treatment, was asked, had she gone back to the UK: ‘do you think that would have been better?’. In working through her reply, Margaret considers carefully the quality of care she has experienced in the Spanish system, citing ‘better treatment here’ and a ‘fantastic, absolutely marvellous’ administrative system at the hospital which meant that X-rays were efficiently ordered and reviewed. Her comparison with the UK is again speculative:

I think if you go for an X-ray in England you can be sitting around waiting for hours … and I think people in England were then waiting for up to 2 years for a hip operation. (Margaret)

With little recent experience to draw on, rationales for distrusting the British system typically therefore cited memories, speculations or media accounts, which had apparently dented background confidence. Few participants reported recent experiential encounters with the UK NHS that had provided opportunities for the ‘faith’ elements of interpersonal trust to be fostered. Peter, tellingly, can utilise his experiential knowledge of care in the Spanish system whereas he can only draw on abstract knowledge of ‘the system’ (‘drivers’) in the UK:

I wouldn't go to the [British] NHS. My experience of medical care is good [in Spain] … The biggest difference about Spain and England is that people care about nursing and the people in England they are more worried about targets. (Peter)

In contrast, even where individual encounters within the institutions of Spanish health care had not gone well, experiential evidence of system performance (such as cleanliness, efficiency and modernity) constituted evidence that the health-care institution looked, felt, and smelled like a hospital: in essence, they exemplified everything that the patient expected in a health-care setting, thereby enabling a background level of trust in the system to be maintained.

## Discussion

As in other studies of health-care experiences (see, for instance, Goold and Klipp 2002), the issue of trust came up spontaneously in our interviews with British pensioners living in Spain. We also utilised their implicit and explicit rationales for choosing one particular health-care system to infer the conditions for trust. The advantages of this particular case study for studying the conditions of trust were that the participants had a choice and, in a new field, were reflective about the rationales for that choice. Our initial puzzle was that trust was apparently bestowed even in cases where there appeared to be few grounds for this being a rational calculation of what Gambetta (1988: 217) calls subjective probability. Here was a clear case of the elusive and somewhat esoteric faith element of trust, and (importantly) one that did not seem reducible to either dependence (Meyer and Ward 2013) or the need to minimise ‘migrant regret’. In moving to Spain and encountering an unfamiliar health-care system, habitus was misaligned with field and taken-for-granted assumptions were disrupted, including those relating to how health-care professionals relate to older patients. This had both positive and negative implications: they were treated with more respect and empathy in the encounter, but at the system level, faced with what was considered to be inadequate provision for in-patient nursing or long-term care. However, most participants demonstrated what could properly be called trust in both the system and their individual practitioners, typically choosing them over candidate alternatives. Drawing on Bourdieusian concepts of field and habitus, and Luhmann's concepts of familiarity, confidence and trust, we have outlined some tentative conditions for trust at the level of both the encounter and the system.

Trust in individual encounters related primarily to faith, rather than rational calculations of risks and benefits. This was fostered through interpersonal elements such as the communication of reciprocity, respect and (often embodied) empathy. This corroborates arguments that contemporary health care relies not on a traditional asymmetrical Parsonian model of trust based on deference towards health-care professionals (Scambler and Britten 2001), but a more relational one in which trust has to be earned by clinicians, and earned primarily through the skilled performance of interrelational skills, rather than clinical competence. Indeed, participants typically suggested that their faith in doctors related to features that are likely to reduce power asymmetries. Physical intimacy and sharing stories, for instance, risked reducing professional distance in the relationship. The importance of interpersonal aspects of care in building trust at the level of the encounter has been well documented in a number of other contexts, including primary care (Robb and Greenhalgh 2006), obstetric care (Zadoroznyj 2001) and gynaecology (Brown *et al*. 2011), and this study confirms that these appear to provide a bedrock for trust, at times overriding indicators of lack of clinical competence. Further, we suggest that the new rules of the game in which older people are valued, and in which empathy is embodied, also functioned to frame the new field (the health-care system) as familiar, rather than ‘foreign’, and as one in which the individual could reasonably bestow implicit trust.

The decision to ‘trust’ individual doctors was based, therefore, largely on faith, fostered by interpersonal skills, rather than a rational calculation of probabilities. However, at the system level, the decision to use Spanish, rather than British, health care, was often rendered rather more calculable in participants’ accounts. There were few grounds on which participants could be said to have a background confidence in Spanish health care as an abstract system: they were critical of the public sector in general, and often lacking in knowledge about how the health system specifically worked. Rather, their trust was bestowed in specific institutions: hospitals and clinics that had been personally experienced as meeting symbolically ideal criteria for a health facility, being clean, modern and efficient. This may represent an objective assessment of facilities that are superior to those likely to be accessed in the UK. However, the fact that these decisions to trust Spanish health care were made whether or not there were the grounds for such a rational assessment suggest something more is going on.

Trust appeared resilient in the Spanish system, in which participants had opportunities for trust to be fostered through experiential encounters that nurtured the faith elements. However, for a group with few recent encounters with the UK system (and having perhaps more negative attitudes to the UK public sector than those who had not migrated), trust appears fragile. Their knowledge of the UK NHS was drawn largely from memories, a common stock of what everybody knows and reference to media accounts. The influence of mass media on public trust in institutions has been noted, with one international study, for instance, attributing different levels of public trust in England and Wales, Germany, and the Netherlands to media images (van der Schee *et al*. 2007). For British pensioners living in Spain, then, the comparative distrust of the UK system may reflect the lack of direct experience of the system through access points in health-care institutions. This is consistent with survey findings that suggest that the public utilise negative news stories when rating their poor satisfaction, in contrast to patients using the service, who are typically more satisfied (Edwards 2006).

Others have suggested that once background confidence in a system is fractured, individual positive experiences with health providers may be insufficient to repair it. Calnan and Rowe (2008b), for instance, found that for patients with diabetes, positive experiences with a particular clinician did not affect their opinion of the health service as a whole, which instead reflected issues such as financial mismanagement and poor hygiene that had been widely covered in British media (Calnan and Rowe 2008b). However, even if encounters with individual practitioners may not be a sufficient condition for trust at the system level to be maintained, our analysis suggests it may be a necessary one.

In our study, trust appears to be built on experiential encounters with specific institutions, not by instrumental knowledge of abstract systems. Indeed, there was little evident knowledge of the system in abstract. Direct knowledge, through accessing health-care institutions, fostered background confidence in the system, as well as trust in individual providers. We can conclude, in this case at least, that there is evidence of the importance of the faith element of trust in health care, not only at the level of the personal encounter but also at the level of the system. Although the foundations for trust in the two levels may differ, and even appear to be in conflict, the decision to bestow trust appears to relate at both levels to symbolic indicators of trustworthiness. For the encounter, these relate to rendering the unfamiliar as familiar and therefore in the realm of taken-for-granted trust (or faith). At the system level, confidence is fostered through symbolic and experiential knowledge of indicators of ideal health institutions, including an aesthetic of hygiene.

Brown (2008), in discussing policy remedies for declining public trust in the health-care system in the UK, notes the importance of adequately understanding how the non-rational bases of trust influence the success of such attempts. Attempts to rebuild trust through ever-increasing monitoring and accountability structures may, he suggests, simply reduce confidence in a health-care system, as they open up spaces for anxiety and heighten awareness of risk, without paying attention to the more important relational elements of trust. Our analysis would support this hypothesis and suggests that policy attempts to increase trust through ever more sophisticated systems for and communication of accountability and risk are unlikely to succeed. In the case of British pensioners living in Spain, the relational and symbolic aspects of health care were a necessary condition for trust. The participants in this study may have rendered their decisions as instrumental ones based on assessments of the two systems at the levels of both health providers and institutions. However, the conditions that have enabled trust to be bestowed in this new field have been shown to be deeply rooted in more symbolic and emotional elements usually associated with faith. Further, this case study suggests a tentative hypothesis regarding the relationship of trust in systems and trust in the encounter. That is, when there are no opportunities to foster trust in practitioners during their interactions with patients, it may be difficult to repair broader public trust in the system.
